# Medical specialties and the job market

**DOI:** 10.1590/S1516-31802011000100001

**Published:** 2011-01-06

**Affiliations:** I Associate professor in the Discipline of Thoracic Surgery, Hospital das Clínicas, Faculdade de Medicina, Universidade de São Paulo (HCFMUSP), São Paulo, Brazil.; II Physician and preceptor of the Discipline of Thoracic Surgery, Hospital das Clínicas, Faculdade de Medicina, Universidade de São Paulo (HCFMUSP), São Paulo, Brazil.

Professional choices are the product of both conscious and subconscious motivation. Many determining factors are involved, such as family influence, personal identification with the training course and the social status conferred by the profession. In addition to these factors, there are others of subconscious nature.^1^ It is possible that the fascination that medicine continues to hold for young people is not due solely to issues of a conscious nature. The image and the status that the title of physician confers, which was mainly achieved beginning in the nineteenth century, is likely to still be the strongest reason.^2^ The representation of the prestige and power of medicine, allied with the job market, in which there are difficulties but no unemployment, has the ability to place the profession as a symbol of social ascension.^2^

A study conducted among fifth-year medical students at Universidade Federal de Minas Gerais (UFMG) showed that a majority of the students (55%) chose to study medicine because they identified with the profession (vocation or personal fulfillment). Altruistic motives and the search for knowledge reached 25% and 20%, respectively. Entering the job market was not indicated as an important factor in choosing a profession.^2^ A similar study among sixth-year medical students at Universidade Federal do Rio Grande do Norte (UFRN) identified the following as the main factors in choosing a career: (1) the possibility of financial independence; (2) personal identification with the profession; and (3) altruism. Entering the job market was not cited as a determining factor.^1^

After concluding the medical course, new professionals need to decide whether to sit a competitive examination for medical residence, and the specialty or subspecialty that they intend to follow. The number of places for medical residence varies between institutions and according to the needs of each service. The number of places for any given specialty does not follow the needs of the job market in each locality. Independent of whether there is a lack of certain specialists in a given state or municipality, the number of places for residence in that position is not changed.

The tendency towards specialization, which is excessive in the case of medicine, can be perceived starting from the undergraduate medical course. Only 15.5% of the fifth-year students and 16.7% of interns at UFMG intended to follow a generalist specialty, such as internal medicine (< 2%), general pediatrics (< 5%), general surgery (< 8%) or gynecology and obstetrics (< 5%).^2^

This predisposition occurs not only in Brazil but also in the United States: since the end of the 1980s, the number of American physicians following generalist careers has been declining.^3^ In 1987, 49.2% of the students were planning a career in internal medicine, family medicine or pediatrics. This percentage has been continually falling: to 43.1% in 1991, and reaching its lowest point in 2002, with 21.5%.^3^ Likewise, from a cohort of physicians in the United Kingdom, it was shown that 10 years after graduation, half of the physicians were working in fields other than their specialties, and most of them were working within general or family medicine.^4^

This leads to a question. Students and medical residents increasingly intend to specialize, and reject generalist careers. Nevertheless, there is no space in the job market, and many of these individuals end up working as family physicians, general clinicians, general surgeons and general pediatricians. A survey by the American Association of Cardiothoracic Surgery Residents (www.ctsnet.org) showed that 20% of the physicians concluding their residence in thoracic and cardiovascular surgery did not have employment within the specialty.^5^ Forty-four percent of the individuals accepted their current job because it was the best available, 25% took the job for family reasons and 30% said that it was the only job that they were able to find.^5^ When asked whether the number of cardiothoracic surgeons under training ought to reflect the changes in the job market, 87% of these individuals agreed.^5^

Dorsey et al.^6^ demonstrated that a controllable lifestyle had been a determining factor in choosing a profession among 55% of the students of the students at American medical schools. Specialties like internal medicine were passed over because of the uncontrollable life-style, excessive working hours and patients with multiple comorbidities and low expectations of improvement.^6^ A similar example can be seen in Brazil. A survey by the Brazilian Association of Cardiovascular Surgery Residents^7^ found that out of 321 places available for residence and training in cardiovascular surgery in Brazil in 2009, only 53 (16.51%) were filled.

In 2009, the National Support Program (*Pró-Residência*) for training medical specialists in strategic areas for the Brazilian National Health System (*Sistema Único de Saúde*; SUS) was instituted.^8^ The aim of the program is to promote training for physicians in priority specialties and regions that are defined through joint agreement with SUS managers. The stimulus is provided through granting bursaries to residents in medical residence programs that have been accredited and are seeking to expand. Bursaries will also be granted for new medical residence programs that demonstrate that opening the program depends solely on bursa-ries for the residents. The program includes basic fields such as internal medicine, general surgery, pediatrics and gynecology and obstetrics; priority fields such as family and community medicine, psychiatry, geriatrics, clinical and surgical cancerology, radiotherapy, anesthesiology, pathology, intensive medicine, neurology, neurosurgery and orthopedics and traumatology; and the activity fields of neonatology, child psychiatry, trauma surgery and emergency medicine.

According to data from the National Medical Residence Commission, there are currently around 27,000 places for medical residence, which are available for residents in all years.^9^ For first-year residents (R1), there are around 7,000 places. Nonetheless, there is a very large disparity in the competition for positions. While residence in specialties like dermatology and plastic surgery have an excessive number of candidates, places for specialties like family and community medicine (regarded by the government as having prime importance) are remaining unfilled. The job market dictates the ways in which professionals enter it. However, this creates an imbalance in relation to the country's needs.

There is a need for careful assessment of the real requirements of the Brazilian job market. Opening places for priority specialties constitutes a valid strategy, but programs to stimulate and perpetuate the deficit specialties in different localities are needed, in association with a career plan that allows young professionals to stay within their field of activity after concluding their training.
